# A Novel Class-Wise Dynamic Anchor Weighting Fusion Framework for Cross-Condition Fault Diagnosis of Offshore Wind Turbine Drivetrain Systems

**DOI:** 10.3390/s26196060

**Published:** 2026-09-24

**Authors:** Pude Li, Yingjie Zhang, Biliang Lu, Feng Ma, Jiongming Yang

**Affiliations:** 1 College of Computer Science and Electronic Engineering, Hunan University, Changsha 410082, China; lipude@hnu.edu.cn (P.L.); zhangyj@hnu.edu.cn (Y.Z.); 2 State Key Laboratory of Maritime Technology and Safety, Wuhan University of Technology, Wuhan 430070, China; martin7wind@whut.edu.cn; 3 Research and Development Center, Goldwind Science & Technology Co., Ltd. (Goldwind), Beijing 100176, China; yangjongming@goldwind.com

**Keywords:** offshore wind turbine, drivetrain system, fault diagnosis, multi-source domain adaptation, class-wise dynamic weighting fusion

## Abstract

Offshore wind turbines operate under harsh and time-varying marine environments, leading to significant distribution shifts between source and target domains. These challenges are further intensified by limited fault samples, imbalanced class distributions, and difficult maintenance access in maritime scenarios. To address these issues, this paper proposes a multi-source domain adaptation method, termed the class-wise dynamic anchor weighting fusion framework (CDAWF), for offshore wind turbine drivetrain fault diagnosis. CDAWF dynamically assigns weights to different source domains according to their feature-level similarity to the target domain, enabling adaptive feature fusion across operating conditions. Convolutional neural networks are used to extract domain-specific representations, while adversarial training is introduced to reduce cross-domain discrepancies. The proposed method is validated on a multi-condition bearing fault dataset generated from high-fidelity simulations of a 5 MW offshore wind turbine drivetrain. Experimental results show that CDAWF effectively improves diagnostic robustness under domain shift, limited labeled data, and class imbalance. Compared with benchmark methods, CDAWF achieves superior classification performance and serves as a useful reference for cross-condition fault diagnosis of offshore wind turbine drivetrain systems.

## 1. Introduction

Wind energy has become an indispensable part of the global transition towards sustainable power generation due to its environmental friendliness, abundant resources, and increasing cost-effectiveness [1]. In recent decades, advances in turbine technology, grid integration, and energy storage have greatly promoted the rapid development of wind power, driving its global cumulative installed capacity to record highs [2]. According to the 2025 Global Wind Report, the cumulative installed global wind power capacity is projected to exceed 1200 GW by 2025 [3].

Offshore wind contributes an increasing proportion of new capacity additions. Despite the benefits of stronger and more stable wind resources, the marine environment is highly complex, imposing greater technical and operational demands on system performance and maintenance [4]. With the rapid deployment of offshore wind power, the long-term reliability, high operational efficiency, and economic sustainability of wind energy systems have emerged as key issues [5].

Within wind turbine systems, the drivetrain is a key subsystem responsible for transmitting mechanical energy from the rotor to the generator [6]. Due to long-term exposure to fluctuating operating conditions, complex dynamic loads, and harsh environmental stresses, the drivetrain is also one of the most vulnerable subsystems of the turbine [7]. Failures of key components, such as gearboxes and bearings, often result in prolonged downtime and substantial maintenance costs. In some wind energy projects, drivetrain faults and associated maintenance activities can account for approximately one-quarter to more than one-third of the total levelized cost of energy [8]. Accurate and early diagnosis of drivetrain faults is essential for improving system reliability, reducing maintenance costs, and preventing severe failures. As wind turbines increase in size and offshore deployment continues to expand, effective drivetrain condition monitoring and fault diagnosis have become increasingly important [9].

Considerable research efforts have been devoted to improving the condition monitoring of wind turbine drivetrain systems. However, reliable and effective fault diagnosis for these systems remains challenging [10]. Two main challenges remain. First, data collected under different operating environments—such as varying wind speeds, turbulence levels, temperatures, or wave loads in offshore systems—tend to exhibit substantially different feature distributions [11]. This phenomenon, commonly referred to as domain shift, significantly undermines the generalization ability of traditional machine learning models, which typically assume that the training and test data follow similar statistical distributions [12]. Second, the availability of labeled samples is often highly imbalanced [13]. In contrast with the large amount of data collected under normal conditions, fault samples, especially those related to incipient faults, are significantly more difficult to obtain due to their low occurrence frequency and the high cost of simulation or collection in real-world operating environments [14]. Therefore, models tend to be biased toward the class with more training samples. This may adversely affect both the accuracy and reliability of fault diagnosis [15].

These challenges are even more pronounced in offshore wind applications [16]. Wind turbines are subjected to multiple interacting environmental loads, which increase the complexity of drivetrain dynamics and result in greater variability in monitoring signals [17]. Moreover, offshore maintenance is difficult and costly due to logistical constraints. Early fault detection is particularly important for reducing unplanned repair costs [18]. These factors highlight the need for an intelligent fault diagnosis method that can adapt to varying operating conditions and effectively utilize highly imbalanced data without compromising diagnostic performance [19].

Various approaches have been investigated to address these challenges. Among them, model-based observer methods have been widely used for fault detection and isolation in wind turbine systems. For example, sliding-mode observers have been applied to fault detection and isolation in wind turbine pitch and drivetrain systems [20], as well as fault estimation and reconstruction in wind turbine main drive systems [21]. These methods rely on system models and observer-based fault estimation.

Recently, large language models (LLMs) and vision-language models (VLMs) have also been explored for intelligent fault diagnosis. Recent studies have investigated adaptive industrial LLMs for mechanical fault diagnosis under variable operating conditions [22], as well as RLHF-optimized industrial VLMs with multimodal learning for fault detection under imbalanced sample distributions [23]. These studies demonstrate the emerging potential of large-model-based approaches for intelligent fault diagnosis in complex industrial scenarios. Among data-driven approaches, domain adaptation (DA) has gained considerable attention in recent fault diagnosis research [24]. By transferring diagnostic information on labeled source domains and narrowing inter-domain distribution gaps, DA enhances the robustness and generalization ability of models under varying operating conditions [25]. Nevertheless, most current studies have been developed under single-source DA settings, whereas practical wind farm monitoring scenarios usually involve multiple heterogeneous domains. These domains often differ significantly in environmental conditions, sensor configurations, and historical fault sample distributions [26]. Therefore, how to achieve collaborative fusion of complementary features across multiple source domains while alleviating the negative transfer caused by less relevant sources has become a key issue in improving the cross-condition drivetrain fault diagnosis [27].

Similarity-based and non-uniform source weighting strategies have been used in existing MSDA methods. However, these strategies generally evaluate source relevance at the domain level. They may therefore overlook differences in source transferability across fault classes. CDAWF instead evaluates source-to-target transferability separately for each fault class using source and target class anchors. Thus, the same source domain can contribute differently to different fault classes. Existing prototype-based methods mainly use class prototypes or centroids to characterize class structure or reduce class-conditional distribution discrepancies. In CDAWF, the class anchors are further used to estimate class-wise source transferability and determine the corresponding source weights. These weights are dynamically updated as the target class anchors evolve during training.

Motivated by the above considerations, this study proposes a novel multi-source domain adaptation framework for wind turbine drivetrain fault diagnosis, referred to as the Class-wise Dynamic Anchor Weighting Fusion Network (CDAWF). The CDAWF framework incorporates three key components to tackle the challenges outlined above: (1) source-specific feature extraction via convolutional neural networks (CNNs), enabling the model to capture domain-dependent fault characteristics from each source; (2) class-wise dynamic anchor weighting, in which source and target class anchors are constructed in the latent feature space and their class-conditional similarities are used to estimate source-to-target transferability. Unlike a single domain-level weighting rule, the contribution of the same source domain can vary across different fault classes, enabling more fine-grained multi-source feature fusion; and (3) domain-adversarial training to reduce cross-domain distribution discrepancies and improve the transferability of the fused feature representation.

To address the above issues, the main contributions of this study are summarized as follows:

(1) This study develops a novel class-wise dynamic anchor weighting fusion framework (CDAWF) for fault diagnosis of offshore wind turbine drivetrains under varying operating conditions, with the aim of handling multi-source distribution shifts and insufficient labeled fault data.
(2) A class-wise dynamic anchor weighting strategy is proposed to evaluate the transferability from source domains to the target domain at the class level. This strategy constructs class anchors for both source and target domains and dynamically generates class-dependent source weights based on their feature similarities, enabling the same source domain to contribute differently to different fault classes. Compared with global source-domain weighting, this approach achieves more fine-grained multi-source fusion and effectively suppresses negative transfer caused by weakly correlated source–class relationships.
(3) Extensive experiments are conducted under multiple transfer scenarios with different domain shifts and class imbalance ratios to systematically evaluate the proposed framework, revealing the joint effectiveness of feature distribution alignment and class-wise weighting in improving diagnostic robustness under varying operating conditions.

The diagnostic performance of CDAWF was evaluated on a multi-condition drivetrain fault dataset derived from a 5 MW offshore wind turbine simulation model under different environmental conditions. Comparisons with several baseline methods demonstrate improved fault classification performance. In addition, the t-SNE visualizations qualitatively illustrate improved class separability and cross-domain feature alignment.

The remainder of this paper is organized as follows. Section 2 presents the proposed CDAWF framework and its main components. Section 3 describes the 5 MW offshore wind turbine drivetrain simulation model and dataset. Section 4 presents and discusses the experimental results. Finally, Section 5 summarizes the main conclusions and discusses future research directions.

## 2. Methodology

### 2.1. Problem Definition

Domain: Let a domain be denoted as D={X,Y}, where X represents the set of acceleration time-series samples obtained by partitioning the raw vibration signals, and Y={1,2,…,C} denotes the shared label space, with *C* being the total number of fault categories. In this study, a multi-source single-target fault diagnosis scenario is considered, involving *M* labeled source domains ${\left\{D_{s}^{\left(i\right)}\right\}}_{i=1}^{M}$ and one unlabeled target domain $D_{t}$. Although all domains share the same label space, their data distributions vary under different environmental conditions.

Problem: In the multi-source unsupervised domain adaptation task for wind turbine drivetrain fault diagnosis, the *i*-th labeled source domain is denoted as $D_{s}^{\left(i\right)}={\left\{\left(x_{n}^{\left(i\right)},y_{n}^{\left(i\right)}\right)\right\}}_{n=1}^{N_{i}}$, where $x_{n}^{\left(i\right)}$ and $y_{n}^{\left(i\right)}$ denote the *n*-th source sample and its corresponding fault label, respectively, and $N_{i}$ is the number of samples in the *i*-th source domain. The total number of labeled source samples is denoted by $N_{s}=\sum_{i=1}^{M}N_{i}$. The unlabeled target domain is denoted as $D_{t}={\left\{x_{m}^{t}\right\}}_{m=1}^{N_{t}}$, where $N_{t}$ is the number of target samples. This task seeks to exploit the complementary information from multiple source domains to improve diagnostic performance on the target domain. Compared with traditional single-source domain adaptation, this task is more challenging because it must simultaneously handle heterogeneous source information, cross-domain distribution discrepancies, and class imbalance.

Task: Given *M* source-specific feature extractors ${\left\{G_{f}^{\left(i\right)}\right\}}_{i=1}^{M}$, a shared label classifier $G_{y}$, and a domain discriminator $G_{d}$, the objective is to learn domain-invariant and class-discriminative representations across multiple source domains and transfer the learned knowledge to the target domain. For notational simplicity in the general optimization objective, $G_{f}$ denotes the overall feature extraction and fusion mapping of the model. The optimization objective is to minimize the target-domain classification risk, formally expressed as

(1) $$\begin{array}{c} \theta^{*}=\arg\min_{\theta }\epsilon_{t}=E_{(x,y)∼Q}\left[L\left(G_{y}\left(G_{f}\left(x\right)\right),y\right)\right], \end{array}$$

where θ denotes the learnable parameters, $\epsilon_{t}$ denotes the target-domain classification risk, and L(·) denotes the classification loss. To improve transferability, the contributions of different source domains are adaptively determined according to their class-wise relevance to the target domain, thereby reducing negative transfer and improving diagnostic robustness.

### 2.2. Class-Wise Dynamic Anchor Weighting Fusion Framework

The proposed Class-wise Dynamic Anchor Weighting Fusion framework (CDAWF) consists of three key components, as illustrated in Figure 1: source-specific feature extractors, a class-wise dynamic weighting module, and a domain-adversarial adaptation module. CDAWF assigns an individual feature extractor to each source domain to capture domain-dependent fault characteristics from segmented vibration signals. Rather than assigning a single contribution weight to each source domain, CDAWF evaluates source-to-target transferability separately for each fault class. Specifically, class anchors are constructed in the latent feature space, where the similarity between source and target anchors is measured to estimate class-wise semantic proximity. Different from prototype-based alignment methods that mainly use class prototypes to characterize class structure or constrain cross-domain feature alignment, the class anchors in CDAWF are further used to estimate class-wise source-to-target transferability and thereby determine the contribution of each source domain to different fault classes. These similarity scores are then normalized into dynamic fusion weights so that highly transferable source domains contribute more to the final representation, whereas distribution-mismatched domains are suppressed. As the learned feature representations and target-domain class anchors are updated during training, the corresponding class-wise similarities and fusion weights are also dynamically updated. In this way, CDAWF can mitigate negative transfer caused by heterogeneous operating conditions and inter-domain imbalance. To further improve cross-domain generalization, the fused representation is fed into a domain-adversarial learning module. The domain discriminator is a classifier designed to differentiate the origin of feature representations, while the feature extraction and fusion modules are optimized to generate features that are both discriminative for fault classification and confusing for domain discrimination. This adversarial learning process promotes a domain-invariant yet class-discriminative latent space, thereby enhancing diagnostic robustness under unseen working conditions.

Assume that there are *M* labeled source domains and one unlabeled target domain:

(2) $$D_{s}^{\left(i\right)}={\left\{\left(x_{n}^{\left(i\right)},y_{n}^{\left(i\right)}\right)\right\}}_{n=1}^{N_{i}},\;i=1,2,\ldots ,M,$$

and

(3) $$D_{t}={\left\{x_{m}^{t}\right\}}_{m=1}^{N_{t}}.$$

Here, $x\in R^{L}$ denotes an input signal segment and y∈{1,2,3,…,C} denotes the class label. For the *i*-th source domain, the source-specific feature extractor is denoted by $G_{f}^{\left(i\right)}\left(\cdot \right)$, and the extracted feature is written as

(4) $$f_{n}^{\left(i\right)}=G_{f}^{\left(i\right)}\left(x_{n}^{\left(i\right)}\right)\in R^{d}.$$

For the target domain, the same extractor produces

(5) $$f_{t,m}^{\left(i\right)}=G_{f}^{\left(i\right)}\left(x_{m}^{t}\right)\in R^{d},\;i=1,\ldots ,M.$$

Here, *d* denotes the dimensionality of the high-level feature representation used for subsequent class-wise fusion, and d=1024 in the present implementation. Before class-wise dynamic source weighting, the outputs of all source-specific feature extractors are equally aggregated to construct a preliminary representation:

(6) $$\bar{f}\left(x\right)=\frac{1}{M}\sum_{i=1}^{M}G_{f}^{\left(i\right)}\left(x\right).$$

For a target sample $x_{m}^{t}$, the corresponding preliminary representation is therefore $\bar{f}\left(x_{m}^{t}\right)$. Based on this preliminary representation, the corresponding preliminary class probabilities are obtained through the shared classifier $G_{y}$:

(7) $${\tilde{\pi }}_{c}\left(x\right)={\left[\mathrm{softmax}\left(G_{y}\left(\bar{f}\left(x\right)\right)\right)\right]}_{c},\;c=1,2,\ldots ,C.$$

Equation (7) applies to both source and target samples. To characterize class-conditional distributions, the class-wise anchor of class *c* in the *i*-th source domain is defined as

(8) $$a_{s,c}^{\left(i\right)}=\frac{1}{|D_{s,c}^{\left(i\right)}|}\sum_{\left(x,y\right)\in D_{s,c}^{\left(i\right)}}G_{f}^{\left(i\right)}\left(x\right),$$

where

(9) $$D_{s,c}^{\left(i\right)}=\left\{\left(x,y\right)\in D_{s}^{\left(i\right)}:y=c\right\}.$$

At the beginning of training, the source class anchors are initialized as unavailable. Once samples of class *c* appear in the mini-batch of the *i*-th source domain, the corresponding source anchor $a_{s,c}^{\left(i\right)}$ is initialized and updated according to Equation (8). If class *c* is absent from a subsequent source mini-batch, the corresponding source anchor is not updated and its most recently available value is retained.

Since the target domain is unlabeled, its class-wise anchor is estimated using pseudo-labels obtained from the preliminary class probabilities defined in Equation (7). The pseudo-label of a target sample $x_{m}^{t}$ is assigned as

(10) $${\hat{y}}_{m}^{t}=\arg\max_{c}{\tilde{\pi }}_{c}\left(x_{m}^{t}\right).$$

Each target sample is assigned to the class with the highest preliminary class probability. The pseudo-labeled target subset corresponding to class *c* is defined as

(11) $${\hat{D}}_{t,c}=\left\{x_{m}^{t}\in D_{t}:{\hat{y}}_{m}^{t}=c\right\}.$$

The target class anchor associated with the *i*-th source-specific feature extractor is then defined as

(12) $$a_{t,c}^{\left(i\right)}=\frac{1}{\left|{\hat{D}}_{t,c}\right|}\sum_{x\in {\hat{D}}_{t,c}}G_{f}^{\left(i\right)}\left(x\right).$$

At the beginning of training, no target class anchor is assumed to be available, and the class-wise source weights are initialized uniformly as $w_{c}^{\left(i\right)}=1/M$. Once pseudo-labeled target samples of class *c* become available, the corresponding target class anchor is initialized according to Equation (12). If no target sample is assigned to class *c* in a subsequent mini-batch, the target anchor of that class is not updated and its most recently available value is retained. The class-wise similarity and source weights are updated only when the required source and target anchors are available; otherwise, the corresponding source weights retain their current values.

The class-conditional similarity between the *i*-th source domain and the target domain is measured by cosine similarity:

(13) $$s_{c}^{\left(i\right)}=\frac{{\left(a_{s,c}^{\left(i\right)}\right)}^{⊤}a_{t,c}^{\left(i\right)}}{{∥a_{s,c}^{\left(i\right)}∥}_{2}\;{∥a_{t,c}^{\left(i\right)}∥}_{2}}.$$

The similarities are normalized through a temperature-scaled softmax:

(14) $$w_{c}^{\left(i\right)}=\frac{\exp(\alpha s_{c}^{\left(i\right)})}{\sum_{j=1}^{M}\exp\left(\alpha s_{c}^{\left(j\right)}\right)},$$

where α>0 controls the sharpness of the weight distribution.

The reliability of the target class anchor is affected by the quality of the pseudo-labels. Incorrect pseudo-labels may bias the target anchor in Equation (12), which can subsequently affect the class-wise similarity estimation in Equation (13) and the source weights in Equation (14). Since the pseudo-labels and target anchors are iteratively updated during training, the resulting similarity estimates and source weights are adjusted accordingly as the target predictions evolve.

For a sample *x*, the class-aware fused representation is defined as

(15) $$f_{fuse}^{\left(c\right)}\left(x\right)=\sum_{i=1}^{M}w_{c}^{\left(i\right)}G_{f}^{\left(i\right)}\left(x\right).$$

To obtain a unified feature for classification, the class-aware representations are further aggregated according to the preliminary class probabilities:

(16) $$f_{fuse}\left(x\right)=\sum_{c=1}^{C}{\tilde{\pi }}_{c}\left(x\right)f_{fuse}^{\left(c\right)}\left(x\right).$$

where ${\tilde{\pi }}_{c}\left(x\right)$ denotes the preliminary class probability of class *c* defined in Equation (7). The preliminary class probabilities are computed before class-wise dynamic fusion and are therefore independent of the final fused representation. At each training iteration, the preliminary class probabilities and target pseudo-labels are first recomputed using the current network parameters. The available target class anchors are then updated according to Equation (12), followed by recalculation of the class-wise similarities and source weights according to Equations (13) and (14), respectively.

The classification loss is defined on fused source features as

(17) $$L_{\mathrm{cls}}=\sum_{i=1}^{M}\frac{1}{N_{i}}\sum_{n=1}^{N_{i}}\ell_{\mathrm{ce}}\;\left(G_{y}\left(f_{fuse}\left(x_{n}^{\left(i\right)}\right)\right),y_{n}^{\left(i\right)}\right).$$

To reduce domain discrepancy, the adversarial domain loss is defined as

(18) $$\begin{array}{cc} L_{\mathrm{adv}}= & \;\frac{1}{N_{s}}\sum_{x\in D_{s}}\ell_{\mathrm{bce}}\;\left(G_{d}\left(f_{fuse}\left(x\right)\right),1\right)\\ \; & +\frac{1}{N_{t}}\sum_{x\in D_{t}}\ell_{\mathrm{bce}}\;\left(G_{d}\left(f_{fuse}\left(x\right)\right),0\right). \end{array}$$

To enhance the intra-class compactness of labeled source features, a center regularization term is introduced as

(19) $$L_{\mathrm{ctr}}=\frac{1}{N_{s}}\sum_{i=1}^{M}\sum_{n=1}^{N_{i}}{∥f_{fuse}\left(x_{n}^{\left(i\right)}\right)-c_{y_{n}^{\left(i\right)}}∥}_{2}^{2}.$$

where $c_{y_{n}^{\left(i\right)}}$ denotes the feature center corresponding to the ground-truth class $y_{n}^{\left(i\right)}$ of the *n*-th sample from the *i*-th source domain. The center regularization is applied only to labeled source samples; no additional center loss is imposed on unlabeled target samples, and target-domain pseudo-labels are not used in this term.

The class center $c_{k}$ is initialized using the mean fused representation of the labeled source samples from the first mini-batch containing class *k*. During training, the class centers are iteratively updated using the labeled source fused representations in the current mini-batch. Specifically, with *B* denoting the batch size for each source domain, the class center $c_{k}$ is updated as

$$\begin{array}{cc} \Delta c_{k} & =\frac{\sum_{i=1}^{M}\sum_{n=1}^{B}I\;\left(y_{n}^{\left(i\right)}=k\right)\left[c_{k}-f_{\mathrm{fuse}}\;\left(x_{n}^{\left(i\right)}\right)\right]}{1+\sum_{i=1}^{M}\sum_{n=1}^{B}I\;\left(y_{n}^{\left(i\right)}=k\right)},\\ c_{k} & \leftarrow c_{k}-\Delta c_{k}. \end{array}$$

where I(·) denotes the indicator function. If class *k* is absent from the current source mini-batch, its center remains unchanged in that iteration.

It should be noted that the class centers used in Center Loss are distinct from the source and target class anchors used for class-wise source weighting. The Center Loss centers are maintained in the unified fused feature space and are updated only using labeled source samples to promote intra-class feature compactness. In contrast, the source and target class anchors are constructed separately in each source-specific feature space and are used to estimate class-wise source-to-target similarities and determine the corresponding source weights. Target pseudo-labels are used only for constructing the target class anchors and are not involved in Center Loss.

Accordingly, the overall objective is formulated as

(20) $$L\left(\theta_{f},\theta_{y},\theta_{d}\right)=L_{\mathrm{cls}}+\beta L_{\mathrm{ctr}}-\lambda L_{\mathrm{adv}},$$

where λ>0 and β>0 are balance coefficients.

The minimax optimization problem is written as

(21) $$\left(\theta_{f}^{*},\theta_{y}^{*}\right)=\arg\min_{\theta_{f},\theta_{y}}L\left(\theta_{f},\theta_{y},\theta_{d}^{*}\right),$$

and

(22) $$\theta_{d}^{*}=\arg\max_{\theta_{d}}L\left(\theta_{f}^{*},\theta_{y}^{*},\theta_{d}\right).$$

Equivalently, when a gradient reversal layer is used, the discriminator minimizes $L_{\mathrm{adv}}$, while the feature extractors are updated in the opposite direction to maximize domain confusion. The iterative updates can be expressed as

(23) $$\theta_{f}\leftarrow \theta_{f}-\mu \left(\frac{\partial L_{\mathrm{cls}}}{\partial \theta_{f}}+\beta \;\frac{\partial L_{\mathrm{ctr}}}{\partial \theta_{f}}-\lambda \;\frac{\partial L_{\mathrm{adv}}}{\partial \theta_{f}}\right),$$

(24) $$\theta_{y}\leftarrow \theta_{y}-\mu \;\frac{\partial L_{\mathrm{cls}}}{\partial \theta_{y}},$$

and

(25) $$\theta_{d}\leftarrow \theta_{d}-\mu \;\frac{\partial L_{\mathrm{adv}}}{\partial \theta_{d}},$$

where μ is the learning rate.

After training, inference on new target samples is performed without updating the target class anchors or class-wise source weights. For a new sample *x*, the source-specific feature extractors first generate ${\left\{G_{f}^{\left(i\right)}\left(x\right)\right\}}_{i=1}^{M}$, from which the preliminary representation $\bar{f}\left(x\right)$ and the corresponding class probabilities ${\tilde{\pi }}_{c}\left(x\right)$ are obtained according to Equations (6) and (7). The class-aware representations are then constructed using the class-wise source weights obtained during domain adaptation, and the final fused representation $f_{\mathrm{fuse}}\left(x\right)$ is calculated according to Equation (16). Finally, the predicted class is determined by the shared classifier $G_{y}$. Therefore, the target testing samples are used only for inference and are not involved in updating the class anchors, fusion weights, or model parameters.

For the single-source setting (M=1), Equation (14) degenerates to $w_{c}^{\left(1\right)}=1$. Consequently, Equation (15) becomes $f_{\mathrm{fuse}}^{\left(c\right)}\left(x\right)=G_{f}^{\left(1\right)}\left(x\right)$. Since the preliminary class probabilities satisfy $\sum_{c=1}^{C}{\tilde{\pi }}_{c}\left(x\right)=1$, Equation (16) further reduces to $f_{\mathrm{fuse}}\left(x\right)=G_{f}^{\left(1\right)}\left(x\right)$. Therefore, the class-wise dynamic source weighting mechanism does not perform source selection in the single-source setting, and its role is primarily reflected in multi-source scenarios (M>1).

### 2.3. CDAWF-Based Fault Diagnosis Scheme for Wind Turbine Drivetrain

The proposed CDAWF-based diagnostic framework consists of the following three stages:

(1) Data acquisition and preprocessing: The acceleration signals were generated from a 5 MW offshore wind turbine drivetrain model, and the collected data were divided into multiple labeled source domains and one unlabeled target domain. The raw acceleration signals are segmented into fixed-length segments to standardize the input shape for neural networks. All segments are min-max normalized to remove scale effects and enhance convergence stability during training. For each source domain, fault categories are labeled, whereas the target domain remains unlabeled to represent the unsupervised adaptation setting considered in this study.
(2) Model training: CDAWF is trained using the combined source and target datasets $D_{s}\cup D_{t}$. During training, the model parameters are iteratively optimized using SGD to minimize the overall objective defined in Section 2.2.
(3) Testing: The optimized CDAWF model with parameters $\theta^{*}$ is applied to the testing dataset $D_{t}$ to perform fault diagnosis on offshore wind turbine drivetrains.

Algorithm 1 presents the detailed training procedure of the CDAWF framework.
**Algorithm 1** Training Procedure of CDAWF**Require:** Source domains ${D_{s}^{\left(i\right)}}_{i=1}^{M}$; target domain $D_{t}$; feature extractors ${G_{f}^{\left(i\right)}}_{i=1}^{M}$; shared classifier $G_{y}$; domain discriminator $G_{d}$; training iterations *T*; batch size *B*; temperature α; balance coefficients λ and β**Ensure:** Trained parameters $\theta_{f}$ and $\theta_{y}$ 1:Initialize $\theta_{f}$, $\theta_{y}$, $\theta_{d}$, $w_{c}^{\left(i\right)}=1/M$, and source and target class anchors as unavailable 2:**for** t=1 to *T* **do** 3:   Sample *B* labeled source samples from each $D_{s}^{\left(i\right)}$ and *B* unlabeled target samples from $D_{t}$ 4:   **for** each source domain i=1 to *M* **do** 5:     Extract source and target features using $G_{f}^{\left(i\right)}$ 6:   **end for** 7:   Compute preliminary representations $\bar{f}\left(x\right)$ according to Equation (6) 8:   Compute preliminary class probabilities ${\tilde{\pi }}_{c}\left(x\right)$ according to Equation (7) 9:   Assign target pseudo-labels ${\hat{y}}_{m}^{t}=\arg\max_{c}{\tilde{\pi }}_{c}\left(x_{m}^{t}\right)$10:   **for** each class c=1 to *C* **do**11:     **for** each source domain i=1 to *M* **do**12:        Update $a_{s,c}^{\left(i\right)}$ if source samples of class *c* are available; otherwise retain its latest available value13:        Update $a_{t,c}^{\left(i\right)}$ if target samples of class *c* are available; otherwise retain its latest available value14:        Compute $s_{c}^{\left(i\right)}$ according to Equation (13) if both $a_{s,c}^{\left(i\right)}$ and $a_{t,c}^{\left(i\right)}$ are available15:     **end for**16:     Compute $w_{c}^{\left(i\right)}$ according to Equation (14) if valid similarities are available; otherwise retain the current weights17:     Compute class-aware representation $f_{\mathrm{fuse}}^{\left(c\right)}\left(x\right)$ according to Equation (15)18:   **end for**19:   Compute final fused representation $f_{\mathrm{fuse}}\left(x\right)$ according to Equation (16)20:   Compute $L_{\mathrm{cls}}$, $L_{\mathrm{adv}}$, and $L_{\mathrm{ctr}}$ according to Equations (17)–(19)21:   Update the Center Loss class centers $c_{k}$ using the labeled source fused representations22:   Update $\theta_{f}$, $\theta_{y}$, and $\theta_{d}$ according to Equations (23)–(25)23:**end for**24:**return** $\theta_{f}$, $\theta_{y}$

## 3. 5 MW Reference Offshore Wind Turbine Model

The simulated 5 MW offshore wind turbine drivetrain model referenced in this study was developed by Nejad et al. using SIMPACK [28], and the dataset collected from this model contains one-hour acceleration signals sampled at 200 Hz. The acceleration signals were used at their original sampling rate in the present study, without additional downsampling or resampling, and no additional anti-aliasing filtering was applied during signal preprocessing. The sampling settings follow the different frequency scales involved in the de-coupled simulation framework. The global wind turbine analysis mainly considers environmental excitations from wind and waves, whose dominant frequency content is generally below 2 Hz and can therefore be adequately represented at 10 Hz. By comparison, the detailed drivetrain model involves internal dynamics at relatively higher frequencies, including those associated with gear meshing; therefore, its acceleration responses were sampled at a higher frequency of 200 Hz [29]. The drivetrain model is configured for the NREL 5 MW wind turbine [30] with the OC3 Hywind tower [31]. More detailed descriptions of these wind turbine models can be found in [32].

Although the dataset is generated from a simulated drivetrain model rather than field measurements, the simulation configuration retains several characteristics relevant to practical vibration-based drivetrain condition monitoring. Specifically, the drivetrain is configured according to the NREL 5 MW reference wind turbine, and axial and radial acceleration responses are acquired at multiple drivetrain locations under different environmental and fault conditions. These acceleration signals correspond to the vibration measurements commonly used for real drivetrain condition monitoring.

The reference drivetrain model is designed with a three-stage transmission structure consisting of two planetary stages followed by a single parallel stage, as illustrated in Figure 2, which also presents the three measurement positions in the drivetrain model used to acquire axial (A) and radial (R) acceleration responses. The dataset covers one healthy condition and three fault conditions, as summarized in Table 1. In the simulation model, bearing degradation is represented by reducing the stiffness value of the corresponding bearing [33]. This modeling strategy is based on the fact that bearing degradation is generally accompanied by changes in the mechanical properties and contact conditions of its internal components. Changes in surface deformation and hardness alter the bearing contact characteristics, thereby resulting in a reduction in stiffness. The degradation of bearing stiffness further affects the dynamic response of the drivetrain and is generally manifested by an increased vibration level. Under healthy conditions, each bearing is assigned its corresponding stiffness value. For each fault condition, the corresponding stiffness value of the faulty bearing is reduced from its healthy value to a prescribed degraded value to construct the degraded bearing condition. The corresponding healthy and degraded stiffness values are summarized in Table 2. In the present dataset, only one prescribed degradation level is considered for each fault condition, without further division into multiple fault severity levels. This fault modeling approach primarily represents changes in the drivetrain dynamic response induced by bearing stiffness degradation and does not model periodic impacts associated with localized bearing defects. Such a fault simulation principle has also been adopted in the fault detection study of Dibaj et al. [29]. Based on the drivetrain bearing vulnerability analysis reported by Nejad et al., the dataset used in this study includes faults at three different bearing locations: the main bearing (INP-B), high-speed shaft bearing (HS-A), and low-speed shaft bearing (IMS-PL-A), corresponding to FC1, FC2, and FC3, respectively. Among these bearings, INP-B and HS-A exhibit relatively high failure probabilities [28].

The dataset includes four environmental conditions (ECs), covering the below-rated (EC1–2), rated (EC3), and above-rated (EC4) regions of the turbine power curve. Other parameters are detailed in Table 3. The dataset generated from this simulation process is partially available for public access in [34].

## 4. Results and Discussion

### 4.1. Data Preparation

Following the sensor placement strategy in Nejad et al. [32], acceleration signals from the main bearing and high-speed shaft bearing are selected because of their high sensitivity to early faults. This selection improves fault-related information and helps reduce data dimensionality, which is important for real-time monitoring of multi-megawatt wind turbines.

To characterize both global and local drivetrain dynamics, each one-hour acceleration record is first temporally divided into two mutually exclusive 30 min intervals. The first 30 min (0–1800 s) are used as the training interval, whereas the remaining 30 min (1800–3600 s) are reserved as the independent testing interval. Sliding-window segmentation is then performed independently within each interval using a window length of L=1024 samples (5.12 s) and an overlap of 700 samples (3.5 s). This procedure produces 1108 candidate segments from each 30 min interval, from which 1000 samples per fault class are selected to construct the experimental dataset. Each 1024-point sample is independently normalized using min–max normalization as

$${\tilde{x}}_{j}=\frac{x_{j}-\min\left(x\right)}{\max(x)-\min(x)+\varepsilon },$$

where $x_{j}$ denotes the *j*-th sampling point of the segmented sample *x*, and ε is a small positive constant introduced to avoid division by zero. The minimum and maximum values are calculated independently for each segmented sample.

The acceleration signals are sampled at 200 Hz, corresponding to a Nyquist frequency of 100 Hz; therefore, the analyzed vibration information is limited to the observable frequency range of 0–100 Hz. To identify the fault-related spectral characteristics retained within this range, Figure 3 presents representative power spectral densities (PSDs) of the acceleration signals under the EC3 operating condition, including the healthy state (FC0), main bearing fault (FC1), high-speed bearing fault (FC2), and low-speed bearing fault (FC3). Differences in spectral amplitude and energy distribution can be observed among these conditions within 0–100 Hz, with more pronounced fault-related variations occurring in several frequency bands. In particular, FC1 exhibits a distinct spectral response at approximately 10–15 Hz, while differences among the four conditions are also observed around the dominant spectral component at approximately 20–25 Hz and in other frequency bands. These spectral differences confirm that fault-related frequency-domain information associated with the different drivetrain conditions is retained within the observable frequency range. Segmented samples are subsequently normalized and used to construct the source-domain dataset, the target-domain adaptation subset for model training, and the target-domain testing subset for cross-domain evaluation.

To evaluate the effectiveness of CDAWF in diagnosing faults in an unlabeled target domain using labeled source-domain data, cross-environment and cross-source fault diagnosis scenarios are considered (e.g., EC1 + EC2 → EC3). A particular focus is placed on addressing the data heterogeneity and imbalance challenges that commonly arise in real-world wind turbine operation.

To this end, two distinct experimental cases are constructed for performance validation: Case *I*, an imbalanced single-source dataset, and Case *II*, an imbalanced multi-source dataset. Across the two cases, a total of sixteen domain adaptation tasks are designed, as listed in Table 4. The proposed CDAWF framework is then compared with existing methods to evaluate its robustness and generalization ability.

Case *I*: This experimental setup considers a single labeled source domain and one unlabeled target domain, where the sample sizes across different fault categories are imbalanced. Specifically, EC1 is used as the source domain and EC2 as the target domain. The numbers of FC0, FC1, FC2, and FC3 samples are 1000, 600, 300, and 100, respectively, in both domains, corresponding to a maximum-to-minimum class imbalance ratio of 10:1. This scenario reflects a common challenge in real wind turbine diagnostics, where healthy data are overrepresented, and labeled faulty data are limited due to the rare occurrence of actual failures. This case aims to evaluate the performance of CDAWF in achieving fault diagnosis with minimal supervision as well as limited fault information.

Case *II*: In this more complex multi-source setting, labeled data are drawn from multiple source domains, and inter- and intra-class imbalances are introduced across the source and target domains. Specifically, EC1 and EC2 are used as the source domains, while EC3 is used as the target domain. For each source domain, the numbers of FC0, FC1, FC2, and FC3 samples are 1000, 600, 300, and 100, respectively, whereas the corresponding numbers in the target domain are 500, 300, 150, and 50. Thus, the maximum-to-minimum class imbalance ratio is 10:1 in each domain. Two types of imbalance are considered simultaneously: (a) inter-class imbalance, where different fault categories have unequal sample sizes, and (b) intra-class imbalance, where samples within the same class are unevenly distributed across domains. The model is trained using the labeled source-domain data together with the unlabeled target-domain data and is subsequently evaluated on the independent target-domain testing subset. This setting aims to reflect realistic diagnostic conditions in offshore WTs and evaluate the performance of CDAWF under severe domain and data shift.

### 4.2. Implementation Details

To evaluate the proposed CDAWF framework, a total of 16 cross-environment tasks are constructed in this study, including 6 single-source cross-environment fault diagnosis tasks in Case *I* and 10 multi-source cross-environment fault diagnosis tasks in Case *II*. These tasks are established based on datasets generated by the 5 MW drivetrain simulation system under four different environmental conditions. For each environmental condition, the dataset contains four fault states (FC0, FC1, FC2, and FC3). To comprehensively assess the effectiveness of CDAWF, we compared the proposed method with several representative domain adaptation methods under both single-source and multi-source settings. Configurations and implementations for these baseline methods are outlined below:

(1) Baseline: As a reference, a standard one-dimensional CNN is used, sharing the same backbone as CDAWF. The only change is in the classifier, where the fine-grained classifier in CDAWF is replaced with a conventional softmax layer. This setup keeps the architecture consistent and makes it easier to isolate the effect of domain adaptation, allowing a clearer comparison with a non-adaptive CNN model.
(2) Single-Source Domain Adaptation (SSDA) Methods: Several classical and advanced SSDA techniques are employed, including: RevGrad [35], a domain-adversarial method that minimizes marginal distribution discrepancy through gradient reversal. DAN [36], which matches source and target feature distributions by minimizing Maximum Mean Discrepancy (MMD). JAN [37], which jointly aligns both marginal and conditional distributions. All SSDA methods use the same 1D-CNN architecture as CDAWF for feature extraction, ensuring fair comparison. For RevGrad and DAN, we re-implement the methods using the official settings, while JAN is implemented following the guidelines in the original work.
(3) Multi-Source Domain Adaptation (MSDA) Methods: To evaluate performance under multiple source domain conditions, we include two recent MSDA baselines: M3SDA [38], which aligns both moment statistics and label predictions across all source–target domain pairs. DCTN [39], which employs a category classifier and domain-specific experts to extract transferable knowledge. All MSDA methods share the same feature extractor (1D-CNN), and domain classifiers are implemented accordingly. Hyperparameters for each model are fine-tuned for optimal adaptation performance.
(4) CDAWF (Ours): Our proposed CDAWF model is based on a 1D-CNN backbone described in Table 5, with the key training hyperparameters listed in Table 6. Unlike conventional SSDA and MSDA models, CDAWF integrates a dynamic source weighting mechanism based on feature similarity and performs weighted feature fusion across domains. This allows the model to emphasize more relevant source domains and suppress negative transfer. Hyperparameters are adjusted for each transfer task to handle varying levels of domain shift and data imbalance.

All methods are implemented in Python 3.7 using a consistent setup, including PyTorch v1.13.1, CUDA v11.7, cuDNN v8.0, and scikit-learn v1.0.2. Experiments are run on a Windows 11 machine (Microsoft Corporation, Redmond, WA, USA) with an Intel Core i7-12800HX CPU (Intel Corporation, Santa Clara, CA, USA), 32 GB RAM (Kingston Technology, Fountain Valley, CA, USA), and an NVIDIA RTX 3080 Ti GPU (NVIDIA Corporation, Santa Clara, CA, USA).

### 4.3. Case I: Single-Source Cross-Domain Fault Diagnosis

#### 4.3.1. Classification Accuracy

In this case, we consider cross-environment fault diagnosis under a single-source domain setting and evaluate the capability of the proposed CDAWF framework to address domain shift and sample imbalance.

Case *I* focuses on evaluating model performance under conditions where different fault categories have unequal sample sizes; the raw segmented vibration signals are directly input into CDAWF and five comparison methods, including three SSDA models (RevGrad, DAN, JAN) and two MSDA models (M3SDA, DCTN). Table 7 shows the diagnostic results on the six imbalanced single-source cross-domain tasks. Among the SSDA baselines, RevGrad achieves an average accuracy of 89.00%, while DAN and JAN attain 86.77% and 89.09%, respectively. Among the MSDA baselines, M3SDA achieves an average accuracy of 92.07%, while DCTN achieves 90.79%. In contrast, the proposed CDAWF method significantly outperforms all baselines with an average classification accuracy of 94.94%.

It should be noted that Case *I* involves only one source domain (M=1); therefore, the class-wise dynamic source weighting degenerates to a unit weight and does not perform source selection in this setting. Nevertheless, CDAWF still achieves better diagnostic performance than the compared single-source adaptation methods. This improvement is mainly attributed to the joint optimization of domain-adversarial alignment and class-discriminative center regularization, which promotes source–target feature alignment while enhancing intra-class feature compactness under imbalanced fault distributions. In comparison, the single-source adaptation methods mainly focus on reducing cross-domain distribution discrepancies and may provide insufficient class-level discrimination under severe class imbalance. For M3SDA and DCTN, although they are originally designed for multi-source domain adaptation, their multi-source modeling advantages cannot be fully exploited when only one source domain is available. Therefore, the results of Case *I* primarily demonstrate the effectiveness of the domain adaptation and class-discriminative learning components of CDAWF rather than the class-wise dynamic source weighting mechanism. The contribution of dynamic source weighting is further evaluated under the multi-source settings in Case *II*.

#### 4.3.2. Feature Visualization

To comprehensively assess the cross-environment fault diagnosis performance of the proposed CDAWF framework, a comparative feature visualization is conducted using the t-distributed stochastic neighbor embedding (T-SNE) technique. For the t-SNE visualizations, up to 1000 segmented samples from each source and target domain were selected in fixed dataset order without additional random or class-stratified subsampling. For each method, the 4096-dimensional convolutional feature representations obtained by flattening the 64×64 output of the fourth convolutional block were extracted from the model checkpoint with the best validation performance and L2-normalized before embedding. The source and target features were concatenated and jointly embedded within each panel using sklearn.manifold. TSNE was used with two output dimensions, a perplexity of 30, PCA initialization, an automatically determined learning rate, 1000 iterations, and a fixed random seed of 42. The same t-SNE settings were used for all compared methods. Owing to space constraints, Task 3 is selected as a representative example to demonstrate the visualization results. Specifically, the extracted 4096-dimensional convolutional feature representations are reduced to two dimensions, facilitating a qualitative assessment of feature discriminability and inter-domain alignment.

The t-SNE visualization of the original data is shown in Figure 4a. The visualizations of the output features produced by RevGrad and DAN are presented in Figure 4b,c. Although these models achieve partial alignment across domains, they exhibit limited capability in effectively distinguishing fault categories, particularly under significant distributional shifts. While DAN employs statistical techniques such as maximum mean discrepancy (MMD) to reduce marginal distribution differences, its reliance on single-source information constrains its ability to resolve complex cross-domain variations.

M3SDA and DCTN, shown in Figure 4d,e, respectively, also improve the alignment between the source and target domains. However, since Case *I* contains only one source domain, their multi-source modeling capability cannot be fully exploited in this setting. The learned features still exhibit relatively scattered class boundaries and partial overlap between fault categories, particularly for target-domain samples. The proposed CDAWF model, shown in Figure 4f, produces more compact feature clusters with clearer separation between different fault categories. As discussed in Section 4.3.1, the class-wise dynamic source weighting reduces to a unit weight in the single-source setting and therefore does not contribute to source selection in Case *I*. The improved feature distribution is mainly associated with the combined effects of domain-adversarial alignment and class-discriminative regularization, which reduce the source–target distribution discrepancy while promoting intra-class feature compactness. Consequently, the source and target samples of the same fault category show improved alignment, while different fault categories remain more clearly separated.

Overall, the feature visualizations in Case *I* show that CDAWF achieves a more favorable feature distribution under the single-source cross-domain setting. Compared with the other methods, source and target samples belonging to the same fault category exhibit closer distributions, while the overlap between different fault categories is reduced. These visualization results are consistent with the role of domain-adversarial alignment in reducing the source–target distribution discrepancy and class-discriminative regularization in promoting intra-class compactness and inter-class separability under imbalanced fault distributions.

### 4.4. Case II: Multi-Source Cross-Domain Fault Diagnosis

#### 4.4.1. Classification Accuracy

By analyzing Case *II*, we examine the robustness of fault diagnosis under realistic data imbalance conditions. As described in Section 4.1, this case involves both inter/intra-class imbalance, making it a more challenging and practical scenario for fault diagnosis of offshore wind turbine drivetrains.

Table 8 and Table 9 show the diagnostic accuracy for ten cross-domain tasks under two-source and three-source settings. Traditional single-source domain adaptation (SSDA) methods such as RevGrad, DAN, and JAN all exhibit significant performance degradation. In Case *II*, their average accuracies are 85.54%, 83.96%, and 84.53%, respectively. These methods can effectively address shifts in marginal distributions but remain limited in handling data imbalance, particularly class imbalance and limited fault samples commonly encountered in practical wind turbine applications.

Multi-source domain adaptation (MSDA) methods, such as M3SDA and DCTN, achieve better performance by leveraging information from multiple source domains. In Case *II*, they reach average accuracies of 91.92% and 86.59%. But they usually rely on uniform weighting or predefined rules to combine source domains, which may not fully capture how each source relates to the target. Therefore, their ability to prevent negative transfer from less relevant domains remains limited.

In contrast, our CDAWF framework performs the best on all ten tasks, with average accuracies of 95.11% and 96.11%. This gain comes mainly from its dynamic weighting strategy, which gives more weight to source domains that are more similar to the target. By looking at both domain shifts and class imbalance together, CDAWF learns features that are robust across domains while retaining discriminative fault information, even under severe imbalance conditions.

These results demonstrate the effectiveness of adaptive multi-source fusion under the simulated cross-condition scenarios considered in this study, where heterogeneous and imbalanced data distributions are introduced across different operating conditions. CDAWF achieves improved cross-domain diagnostic performance while mitigating the adverse effects of data imbalance. However, since the current evaluation is based on simulated drivetrain data, further validation using experimental and field monitoring data is required to assess its applicability under actual offshore wind turbine operating conditions.

#### 4.4.2. Feature Visualization

The above results demonstrate the effectiveness of CDAWF for fault diagnosis under imbalanced data conditions. To further investigate the learned feature distributions under domain shift and class imbalance, t-SNE is employed to visualize the extracted features. Specifically, two-dimensional projections of the high-dimensional feature representations are constructed to qualitatively examine the separability of fault categories and the feature distributions across the source and target domains in Case *II*.

Figure 5a depicts the t-SNE visualization of the original data under Task 9. For SSDA methods such as RevGrad and DAN, the feature visualizations in Figure 5b,c show noticeable overlap among fault categories, indicating limited class separability in the two-dimensional projections. Even with domain alignment techniques such as maximum mean discrepancy (MMD) in DAN, the learned features remain insufficiently separated.

Compared with SSDA methods, MSDA approaches such as M3SDA and DCTN make better use of multiple source domains and show improved alignment at the domain level, as seen in Figure 5d,e. However, they often treat all source domains equally or rely on fixed assumptions, which can limit how effectively information from different sources is combined. This is reflected in the visualization through dispersed cluster boundaries and the partial mixing of fault categories, such as FC1 and FC2, which is consistent with the performance degradation observed under class-imbalanced conditions.

By contrast, the CDAWF model, shown in Figure 5f, produces compact, well-separated clusters for all fault classes, even under severe sample imbalance conditions. Its dynamic source weighting and adaptive fusion strategy enable the model to focus on semantically relevant sources while attenuating the impact of disproportionate sample sizes. As a result, the model preserves both inter-class distinctiveness and cross-domain alignment. In particular, fault categories such as FC1 and FC2 remain well separated in the two-dimensional projection, which is consistent with the improved diagnostic performance reported above. Similar t-SNE results for the three-source setting are presented in Figure 6.

Overall, these visualizations provide qualitative evidence of differences in the learned feature distributions among the compared methods under simultaneous domain shift and class imbalance. Combined with the quantitative diagnostic results reported above, the results indicate that CDAWF achieves more effective cross-domain adaptation and more discriminative feature representations through its dynamic class-wise design.

### 4.5. Ablation Study

To investigate the contribution of each component in the proposed CDAWF framework, an ablation study was conducted by progressively removing the dynamic weighting (DW), domain-adversarial learning (ADV), and center regularization (CTR) modules. Because overall accuracy may be dominated by majority classes, balanced accuracy and Macro-F1 are used together with accuracy to evaluate CDAWF under class imbalance. The corresponding results are summarized in Table 10.

When all three components are removed (w/o DW, ADV, CTR), the model achieves an accuracy of 89.2%, a balanced accuracy of 84.8%, and a Macro-F1 score of 84.6%. Introducing the ADV module while excluding DW and CTR increases the classification accuracy to 91.5%, indicating that adversarial feature alignment effectively reduces the distribution discrepancy between the source and target domains. In comparison, the model with CTR but without DW and ADV achieves an accuracy of 90.6%, demonstrating that enhancing intra-class feature compactness also contributes to cross-condition fault discrimination, although its individual contribution is relatively smaller than that of adversarial alignment.

When ADV and CTR are jointly incorporated while dynamic weighting is removed (w/o DW), the accuracy further increases to 92.7%, with a balanced accuracy of 90.4%. This improvement demonstrates the complementary effects of domain-level distribution alignment and class-level feature compactness. Furthermore, replacing the proposed class-wise dynamic weighting strategy with global dynamic weighting (w/o Class-wise DW) yields an accuracy of 93.1%, a balanced accuracy of 92.3%, and a Macro-F1 score of 91.9%. Although global weighting can adaptively adjust the overall contributions of different source domains, it cannot characterize the class-dependent transferability between individual source domains and the target domain.

The complete CDAWF framework achieves the best performance, reaching 95.2% accuracy, 93.1% balanced accuracy, and 93.3% Macro-F1. Compared with the model without dynamic weighting, CDAWF improves these three metrics by 2.5, 2.7, and 4.0 percentage points, respectively. Moreover, compared with global dynamic weighting, the proposed class-wise weighting mechanism provides improvements of 2.1, 0.8, and 1.4 percentage points, respectively. These results demonstrate that the performance improvement in CDAWF does not originate from a single component; instead, it results from the complementary interaction among domain-adversarial alignment, center regularization, and class-wise dynamic source weighting. In particular, the improvements in balanced accuracy and Macro-F1 further indicate that the proposed class-wise weighting mechanism is beneficial for maintaining robust diagnostic performance under imbalanced cross-condition scenarios.

### 4.6. Convergence Performance and Computational Time

When applying these methods to fault diagnosis, two practical aspects merit particular attention: model convergence during training and computational cost. Because modern DL models are often structurally complex and rely on large amounts of vibration data, training can be computationally demanding. While faster hardware can reduce the overall runtime, our focus here is on the relative convergence efficiency of different models. To this end, three indicators are examined: training time, diagnostic accuracy, and convergence speed over iterations. As shown in Table 11, traditional methods such as SVM require much less training time because they do not rely on iterative deep optimization. In contrast, deep adversarial models such as M3SDA and the proposed CDAWF exhibit longer but justified training durations due to their more sophisticated alignment and fusion strategies.

To compare convergence behavior more clearly, Task 9 is taken as an example, and the convergence curves over 100 training iterations are shown in Figure 7. As shown in Figure 7b, RevGrad and DAN exhibit relatively fast convergence, but their performance levels off early on. This suggests they have limited ability to handle both domain shift and class imbalance. DAN in particular uses MMD for distribution alignment, but when the sample distribution is imbalanced, its training becomes less stable. The loss curve fluctuates considerably, indicating difficulty in achieving stable feature alignment under imbalanced sample distributions.

As shown in Figure 7c, M3SDA leverages multiple source domains for feature alignment, which increases the complexity of balancing information across heterogeneous domains and results in relatively slower convergence. Although M3SDA eventually achieves satisfactory diagnostic performance, its fixed fusion strategy cannot adaptively account for differences in source-to-target relevance.

In contrast, as shown in Figure 7c, CDAWF converges rapidly and remains stable throughout the iterations. This behavior is mainly attributed to its dynamic weighting strategy, which assigns higher weights to source domains that are more similar to the target domain in the feature space.

## 5. Conclusions

Wind turbines under varying environmental conditions produce highly heterogeneous vibration signals, which weaken the generalization ability of conventional fault diagnosis models. This issue is further aggravated by class and domain imbalance, especially in offshore wind farms characterized by harsh environments and unstable sensing conditions. To address this problem, this study proposes CDAWF, a multi-source domain adaptation framework with dynamic source weighting. By emphasizing source domains that are more similar to the target domain, CDAWF improves multi-source feature fusion and source–target alignment under imbalanced conditions. Experiments on a simulated 5 MW offshore wind turbine drivetrain dataset demonstrate that CDAWF outperforms existing single-source and multi-source baselines in cross-environment fault diagnosis. The proposed method effectively mitigates negative transfer and improves generalization to unseen conditions.

Although CDAWF shows promising performance in offshore wind turbine fault diagnosis, several limitations remain. First, the current validation is mainly based on simulated drivetrain data, and the conclusions are therefore limited to the investigated simulation-based cross-condition fault classification and domain adaptation setting. Further experimental and field validation is required to assess the generalization of CDAWF under actual offshore operating conditions. Transfer of the proposed framework to real wind turbine measurements requires further evaluation of sensor-channel and measurement-bandwidth compatibility, preprocessing consistency, the correspondence between simulated fault conditions and real fault mechanisms, and the distribution discrepancy between simulated and measured data. Moreover, the current shared-label formulation assumes that the target fault categories are represented in the source label space. These conditions have not been validated by the present results. Second, although CDAWF shows good performance under the class- and domain-imbalanced scenarios considered in this study, its performance may still degrade when target-domain samples are extremely scarce or fault class distributions are highly skewed. Future work will therefore focus on validation using experimental test-rig data and long-term offshore field monitoring data, together with further robustness evaluation under more severe data imbalance, measurement noise, and sensor uncertainty.

## Figures and Tables

**Figure 1:**
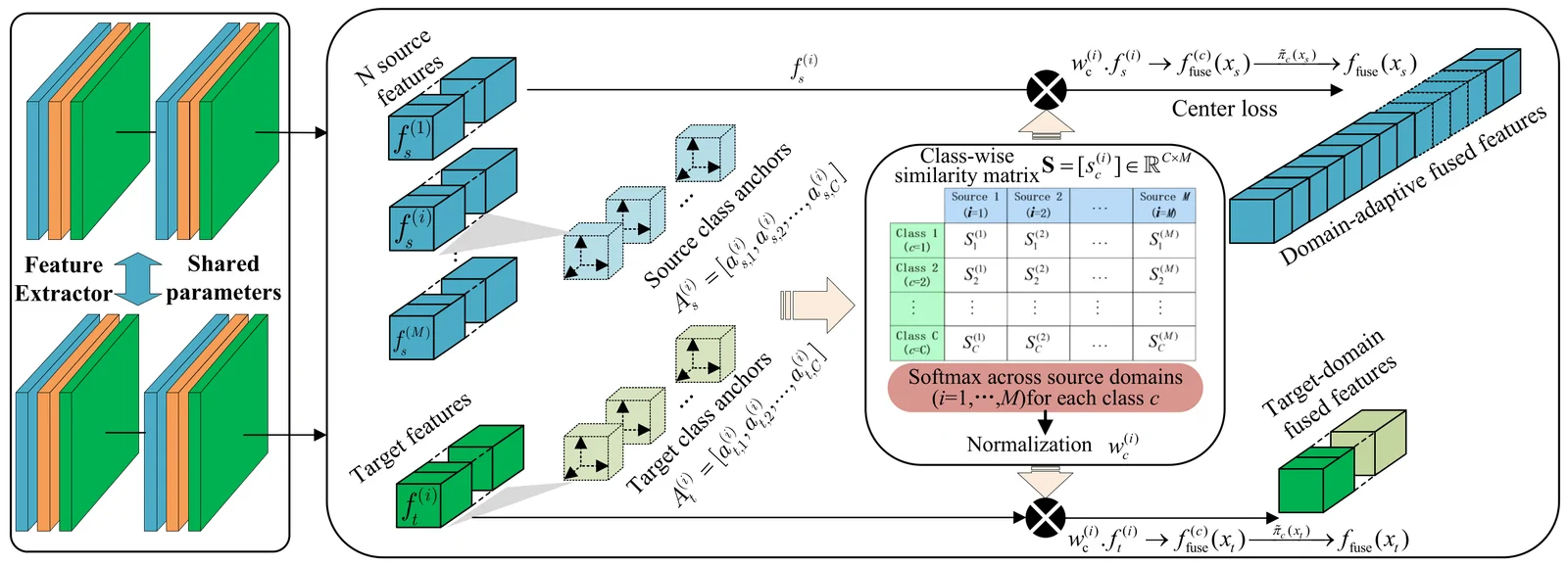
The proposed class-wise dynamic anchor weighting fusion framework.

**Figure 2:**
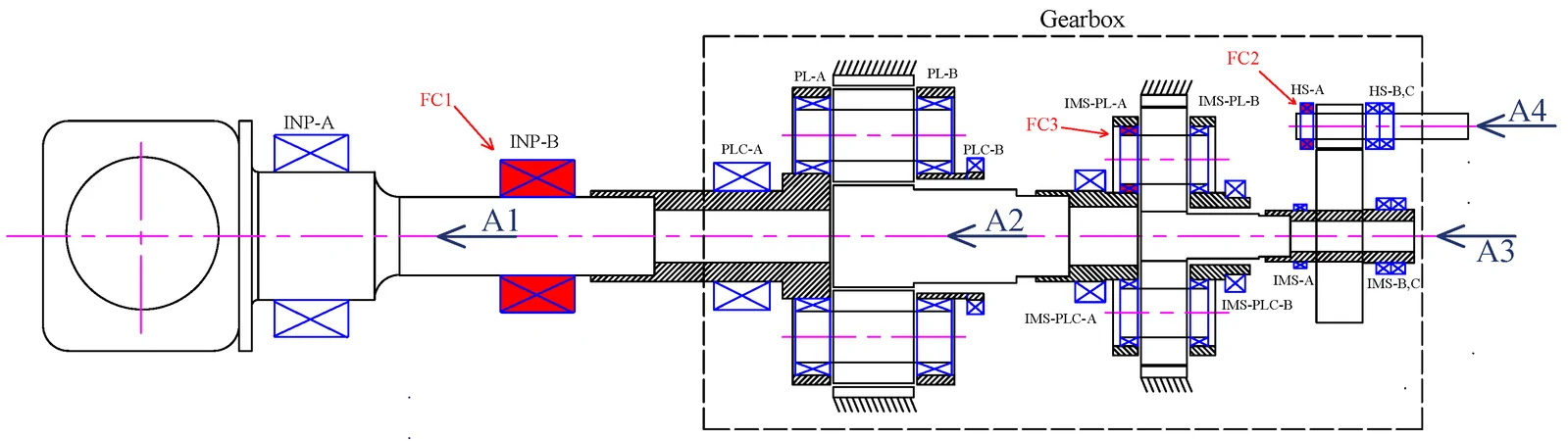
Fault and measurement locations in the gearbox schematic layout.

**Figure 3:**
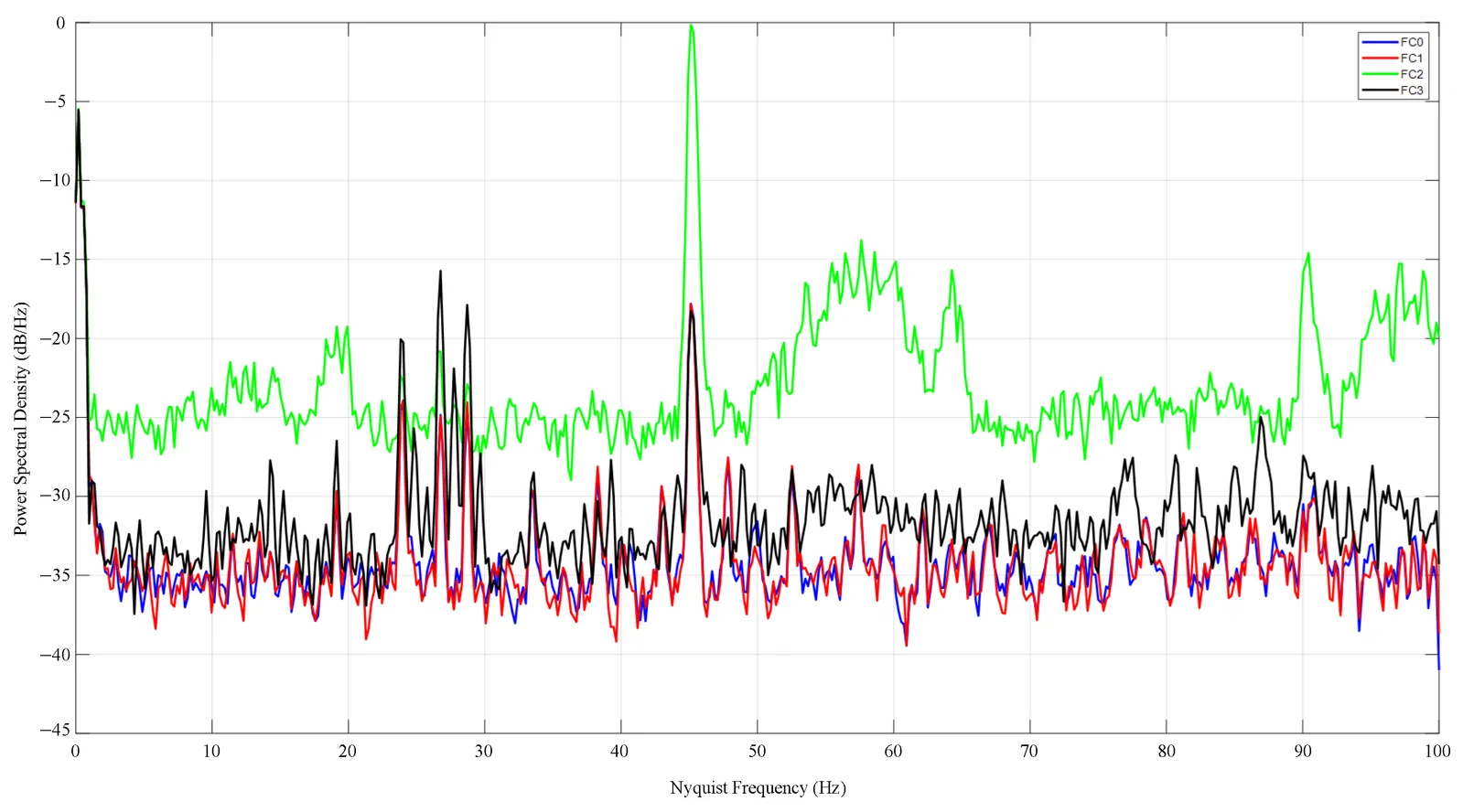
Representative power spectral densities of the acceleration signals under different fault conditions in EC3.

**Figure 4:**
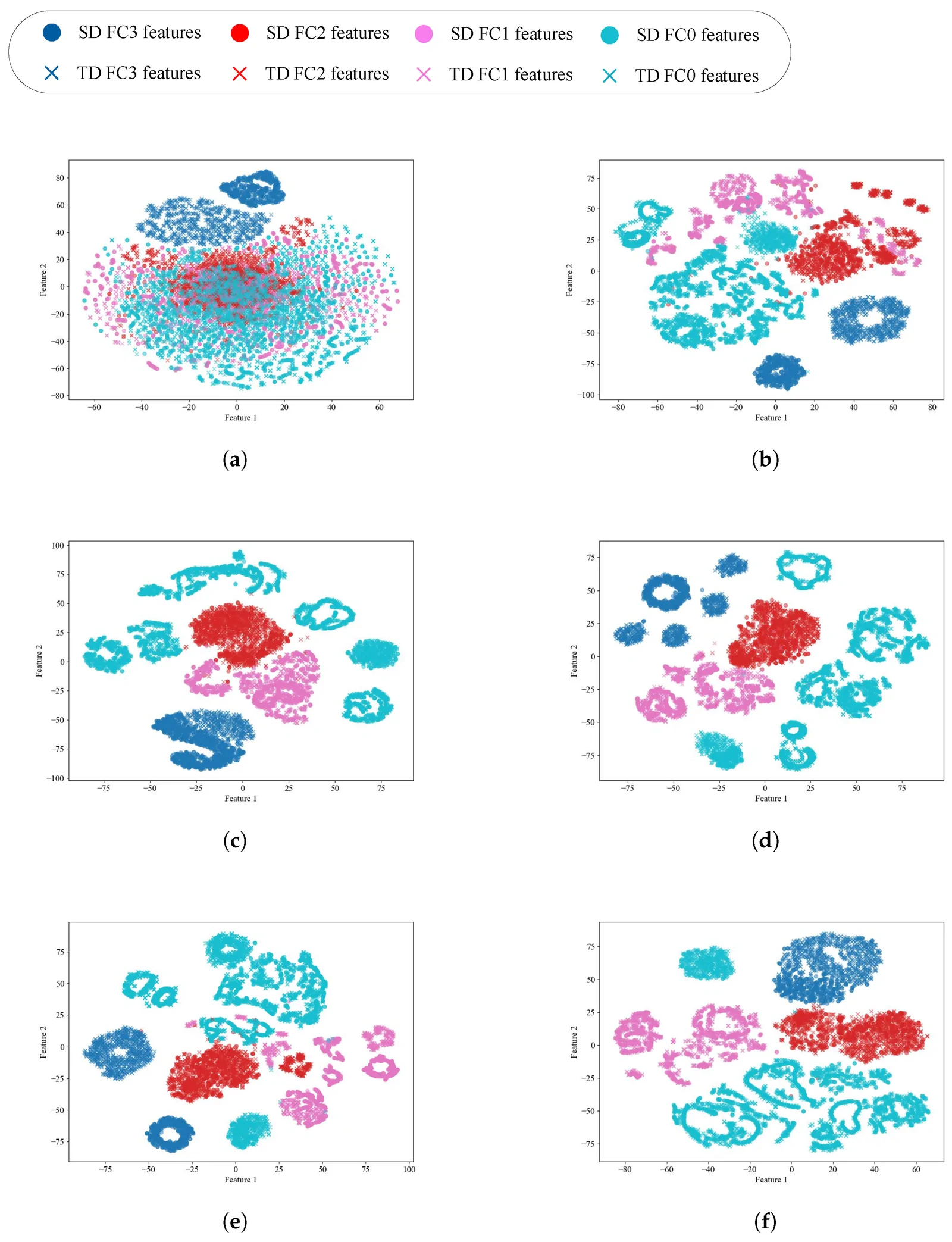
T-SNE visualizations of features in Case *I*, Task 3: (**a**) Original data, (**b**) RevGrad, (**c**) DAN, (**d**) M3SDA, (**e**) DCTN, and (**f**) the proposed method.

**Figure 5:**
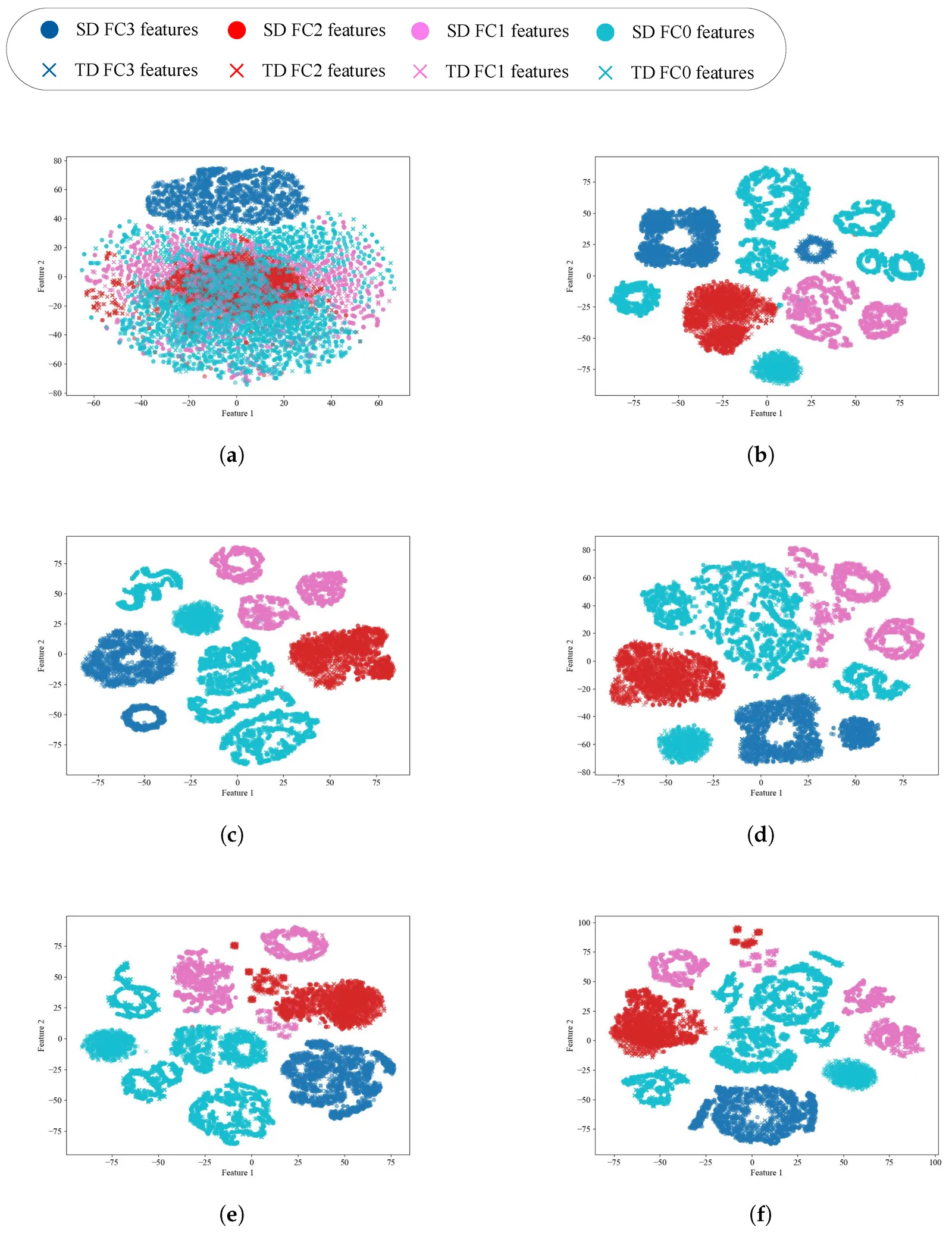
T-SNE visualizations of features in Case *II*, Task 9: (**a**) Original data, (**b**) RevGrad, (**c**) DAN, (**d**) M3SDA, (**e**) DCTN, and (**f**) the proposed method.

**Figure 6:**
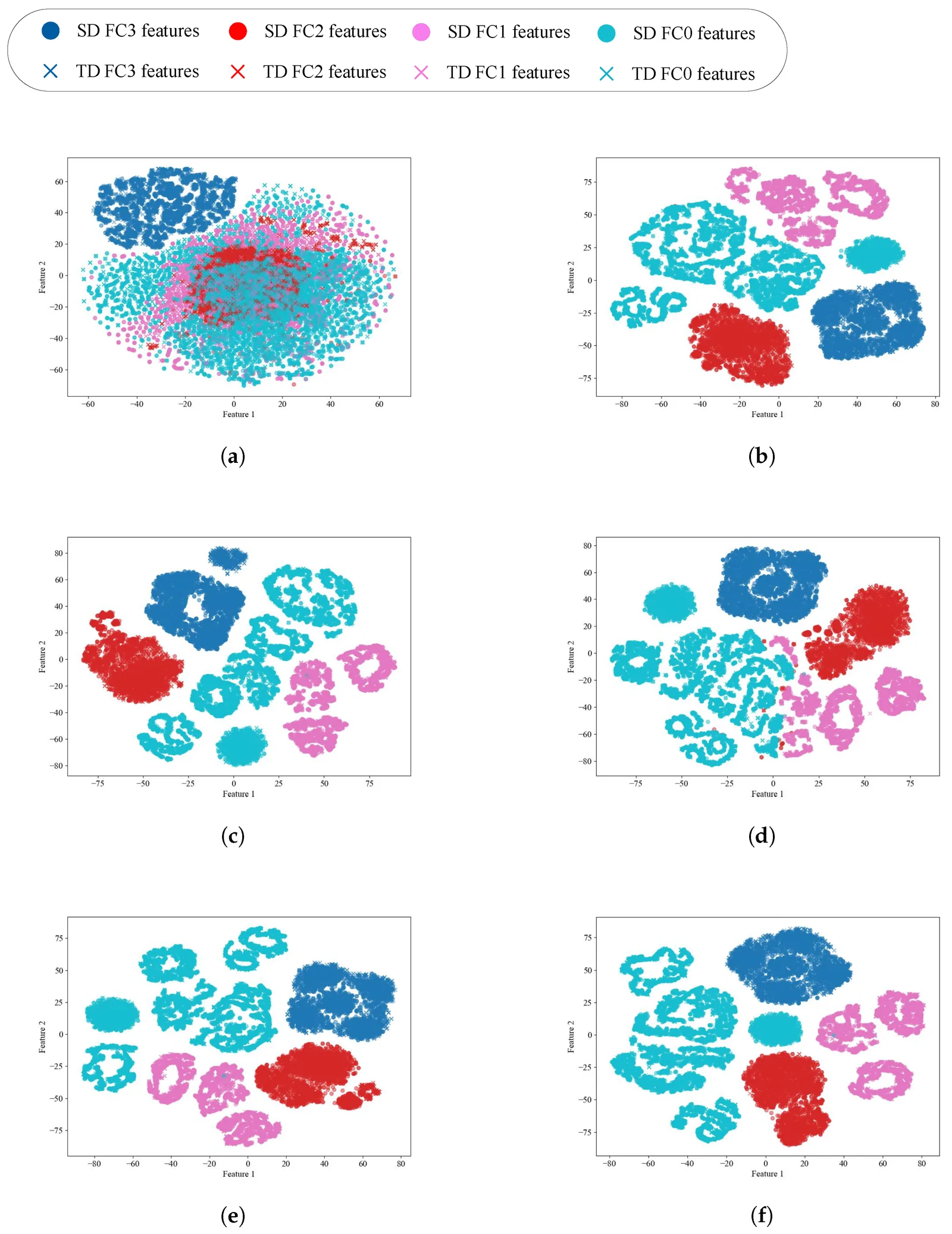
T-SNE visualizations of features in Case *II*, Task 14: (**a**) Original data, (**b**) RevGrad, (**c**) DAN, (**d**) M3SDA, (**e**) DCTN, and (**f**) the proposed method.

**Figure 7:**
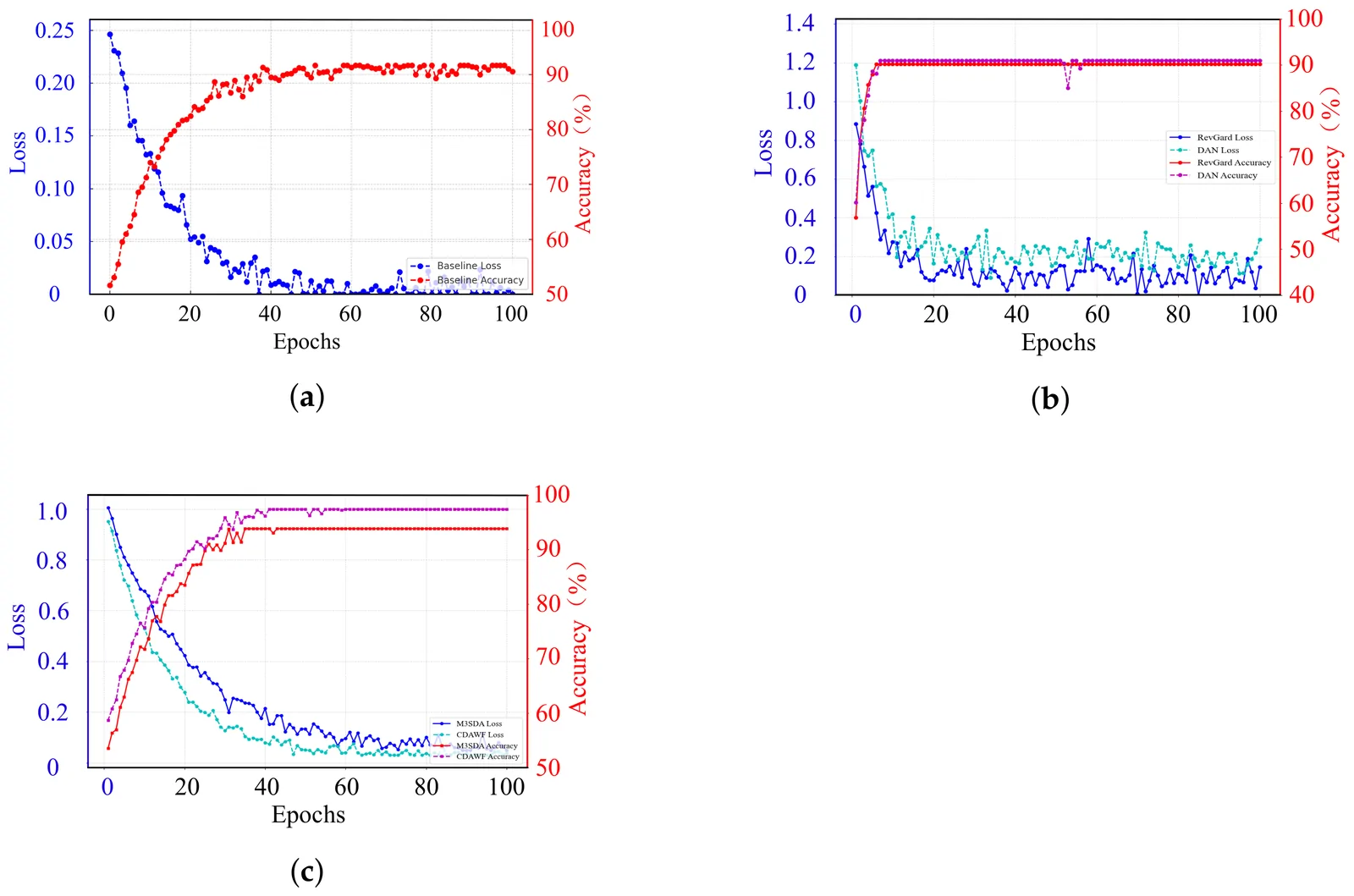
Convergence and accuracy curves of the five methods: (**a**) baseline method; (**b**) RevGrad and DAN; (**c**) M3SDA and the proposed CDAWF. The accuracy curves are calculated on the independent target-domain testing set, while the loss curves represent the total training loss of each corresponding method.

**Table 1:** Fault cases.

Fault Case	Description
FC0	Healthy
FC1	Main bearing fault (INP-B)
FC2	high-speed bearing fault (HS-A)
FC3	low-speed bearing fault (IMS-PL-A)

**Table 2:** Bearing stiffness values for different fault conditions.

Fault Case	Healthy Stiffness Value (N/m)	Reduced Stiffness Value (N/m)
FC0	–	–
FC1	$4.06\times 10^{8}$	$4.06\times 10^{7}$
FC2	$8.2\times 10^{8}$	$8.2\times 10^{6}$
FC3	$6.12\times 10^{7}$	$6.12\times 10^{5}$

**Table 3:** Environmental conditions.

	EC1	EC2	EC3	EC4
Wind speed *U* (m/s)	4.0	7.0	12.0	20.0
Turbulence intensity *I* (−)	0.26	0.19	0.15	0.12
Significant wave height $H_{s}$ (m)	2.0	4.5	5.0	5.5
Spectral peak period $T_{p}$ (s)	8.0	12.0	12.0	14.0

**Table 4:** Sixteen domain adaptation fault diagnosis task setups.

Task	Case	Source	Target	Feature Shift
1	EC1 → EC2	below-rated	below-rated	Slight
2	EC1 → EC3	below-rated	rated	Moderate
3	EC1 → EC4	below-rated	above-rated	Large
4	EC2 → EC3	below-rated	rated	Moderate
5	EC2 → EC4	below-rated	above-rated	Large
6	EC3 → EC4	rated	above-rated	Moderate
7	EC1 + EC2 → EC3	below-rated	rated	Moderate
8	EC1 + EC2 → EC4	below-rated	above-rated	Large
9	EC2 + EC3 → EC1	-	below-rated	-
10	EC2 + EC3 → EC4	-	above-rated	-
11	EC3 + EC4 → EC1	-	below-rated	-
12	EC3 + EC4 → EC2	-	below-rated	-
13	EC1 + EC2 + EC3 → EC4	-	above-rated	-
14	EC1 + EC2 + EC4 → EC3	-	rated	-
15	EC1 + EC3 + EC4 → EC2	-	below-rated	-
16	EC2 + EC3 + EC4 → EC1	-	below-rated	-

**Table 5:** Detailed configuration of one-dimensional CNN.

Layer	Type	R − C − S − P	Output
Input	Data	-	1024 × 1
Block1-conv1	Convolution	64 × 1 − 16 − 1 − 32	1024 × 16
Block1-pool1	MaxPooling	2 × 1 − 1 − 2	512 × 16
Block1-BN1	Batch normalization	-	512 × 16
Block1-ReLU1	ReLU	-	512 × 16
Block2-conv2	Convolution	5 × 1 − 32 − 1 − 2	512 × 32
Block2-pool2	MaxPooling	2 × 1 − 1 − 2	256 × 32
Block2-BN2	Batch normalization	-	256 × 32
Block2-ReLU2	ReLU	-	256 × 32
Block3-conv3	Convolution	5 × 1 − 64 − 1 − 2	256 × 64
Block3-pool3	MaxPooling	2 × 1 − 1 − 2	128 × 64
Block3-BN3	Batch normalization	-	128 × 64
Block3-ReLU3	ReLU	-	128 × 64
Block4-conv4	Convolution	5 × 1 − 64 − 1 − 2	128 × 64
Block4-pool4	MaxPooling	2 × 1 − 1 − 2	64 × 64
Block4-BN4	Batch normalization	-	64 × 64
Block4-ReLU4	ReLU	-	64 × 64
Flatten	Flatten	–	4096
Fc1	Fully-connected	1 × 1 × 1024	1024
Fc2	Fully-connected	1 × 1 × 1024	1024
Output	Fully-connected	1 × 1 × C	C

**Table 6:** Key hyper-parameters of CDAWF for Cases *I* and *II*.

Hyper-Parameter	Case *I*	Case *II*
Learning rate μ	$3\times 10^{-2}$	$1\times 10^{-2}$
Batch size	64	128
α	3	3
β	0.01	0.01
λ	0.1	0.1

**Table 7:** Classification accuracy (%) on imbalanced single source cross-domain tasks.

Methods	Task 1	Task 2	Task 3	Task 4	Task 5	Task 6	Average
Baseline	71.95 ± 3.43	73.12 ± 3.21	78.94 ± 6.61	79.74 ± 4.10	91.08 ± 1.30	88.32 ± 0.75	80.53
RevGrad	92.29 ± 2.93	89.15 ± 3.70	83.42 ± 5.10	87.55 ± 2.95	90.20 ± 2.12	91.36 ± 1.94	89.00
DAN	87.04 ± 4.85	86.67 ± 3.90	81.76 ± 3.00	84.89 ± 3.20	91.03 ± 2.65	89.21 ± 4.58	86.77
JAN	89.12 ± 2.95	84.78 ± 6.42	84.63 ± 6.15	88.14 ± 2.87	95.24 ± 1.67	92.61 ± 2.09	89.09
M3SDA	91.05 ± 3.80	93.39 ± 3.03	88.02 ± 1.45	90.64 ± 2.01	95.01 ± 1.09	94.32 ± 1.40	92.07
DCTN	94.10 ± 2.12	93.76 ± 2.85	85.14 ± 2.81	89.44 ± 2.36	93.63 ± 2.40	88.67 ± 2.22	90.79
CDAWF	96.03 ± 1.61	97.12 ± 2.10	90.42 ± 1.98	91.23 ± 1.75	96.98 ± 1.33	97.85 ± 1.24	94.94

**Table 8:** Classification accuracy (%) on imbalanced two-source cross-domain tasks.

Methods	Task 7	Task 8	Task 9	Task 10	Task 11	Task 12	Average
Baseline	67.49 ± 3.12	69.91 ± 2.98	63.00 ± 4.55	77.47 ± 2.31	93.80 ± 1.04	83.72 ± 1.65	75.90
RevGrad	91.12 ± 2.67	89.76 ± 2.45	75.90 ± 3.83	78.65 ± 2.10	92.45 ± 1.96	85.33 ± 2.08	85.54
DAN	75.21 ± 4.10	86.03 ± 3.66	84.50 ± 2.74	84.40 ± 2.85	91.12 ± 1.78	82.47 ± 3.12	83.96
JAN	71.25 ± 3.94	78.91 ± 4.26	86.77 ± 3.02	84.88 ± 3.47	93.92 ± 1.22	90.45 ± 2.33	84.36
M3SDA	92.80 ± 1.90	89.43 ± 2.58	90.51 ± 1.40	89.05 ± 2.25	95.33 ± 1.08	94.37 ± 1.47	91.92
DCTN	89.98 ± 2.18	88.73 ± 2.36	79.80 ± 2.91	84.52 ± 2.61	92.61 ± 1.80	83.90 ± 2.12	86.59
CDAWF	94.02 ± 1.50	92.65 ± 1.74	95.41 ± 1.37	92.55 ± 1.65	97.78 ± 1.10	98.23 ± 1.25	95.11

**Table 9:** Classification accuracy (%) on imbalanced three-source cross-domain tasks.

Methods	Task 13	Task 14	Task 15	Task 16	Average
Baseline	80.88 ± 2.34	80.71 ± 2.67	82.12 ± 3.01	76.84 ± 2.45	80.14
RevGrad	91.67 ± 1.98	92.95 ± 2.31	94.81 ± 1.74	94.08 ± 1.89	93.38
DAN	90.95 ± 2.42	90.88 ± 2.16	93.92 ± 1.86	92.50 ± 2.01	92.06
JAN	90.04 ± 2.23	91.85 ± 2.35	94.60 ± 1.98	93.33 ± 1.87	92.46
M3SDA	93.85 ± 1.94	90.12 ± 2.27	92.77 ± 2.01	91.96 ± 2.14	92.18
DCTN	87.12 ± 2.89	91.35 ± 2.20	86.48 ± 2.65	85.97 ± 2.76	87.73
CDAWF	97.42 ± 1.31	97.66 ± 1.15	95.84 ± 1.42	93.53 ± 1.64	96.11

**Table 10:** Ablation study of different components in the proposed CDAWF framework.

Method	Accuracy (%)	Balanced Acc. (%)	Macro-F1 (%)
w/o DW, ADV, CTR	89.2	84.8	84.6
w/o DW, CTR	91.5	87.8	88.9
w/o DW, ADV	90.6	87.5	86.7
w/o DW	92.7	90.4	89.3
w/o Class-wise DW	93.1	92.3	91.9
CDAWF	95.2	93.1	93.3

**Table 11:** Training time on Case *II* (Task 9).

Experimental Method	Training Time (s)	Epochs
Baseline	17.45	100
RevGrad	34.47	100
DAN	32.07	100
M3SDA	37.34	100
CDAWF	39.98	100

## Data Availability

The original contributions presented in this study are included in the article. Further inquiries can be directed to the corresponding author.

## Abbreviations

The following abbreviations are used in this manuscript:

WTs	Wind Turbines
ECs	Environmental Conditions
DA	Domain Adaptation
SD	Source Domain
TD	Target Domain
1D-CNN	One-Dimensional Convolutional Neural Networks
RevGrad	Domain-Adversarial through Gradient Reversal
DAN	Deep Adaptation Networks
JAN	Joint Adaptation Networks
M3SDA	Moment Matching for Multi-Source Domain Adaptation
DCTN	Deep CockTail Network
CDAWF	Class-wise Dynamic Anchor Weighting Fusion
